# Safety of Single Stage Revision Laparoscopic Sleeve Gastrectomy Compared to Laparoscopic Roux-Y Gastric Bypass after Failed Gastric Banding

**DOI:** 10.1007/s11695-020-04975-6

**Published:** 2020-09-18

**Authors:** Michał Janik, Christopher Ibikunle, Ahad Khan, Amir H. Aryaie

**Affiliations:** 1 Bariatric Center of Excellence, Department of surgery, Texas Tech University Health Science Center, Lubbock, TX USA; 2 Department of General, Oncologic, Metabolic and Thoracic Surgery, Military Institute of Medicine, Warszawa, Poland; 3 Medical College of Georgia, Augusta, GA USA; 4 Bariatric and Reflux Center, Georgia SurgiCare, Atlanta, GA USA

**Keywords:** Bariatric surgery, Gastric banding, Revisional surgery, Laparoscopic sleeve gastrectomy, Laparoscopic roux-y gastric bypass, Metabolic and bariatric surgery accreditation and quality improvement program (MBSAQIP)

## Abstract

**Background:**

Reoperation, after failed gastric banding, is a controversial topic. A common approach is band removal with conversion to laparoscopic Roux-Y gastric bypass (LRYGB) or laparoscopic sleeve gastrectomy (LSG) in a single-step procedure.

**Objective:**

This study aimed to assess the safety of revisional surgery to LSG compared to LRYGB after failed laparoscopic adjustable gastric banding (LAGB) based on MBSAQIP Participant User File from 2015 to 2018.

**Methods:**

Patients who underwent a one-stage conversion of LAGB to LSG (Conv-LSG) or LRYGB (Conv-LRYGB) were identified in the MBSAQIP PUF from 2015 to 2017. Conv-LRYGB cases were matched (1:1) with Conv-LSG patients using propensity scoring to control for potential confounding. The primary outcome was all-cause mortality.

**Results:**

A total of 9974 patients (4987 matched pairs) were included in the study. Conv-LRYGB, as compared with conv-SG, was associated with a similar risk of mortality (0.02% vs. 0.06%; relative risk [RR], 0.33; 95% confidence interval [CI], 0.03 to 3.20, *p* = 0.32). Conversion to LRYGB increased the risk for readmission (6.16% vs. 3.77%; RR, 1.63; 95%CI, 1.37 to 1.94, *p* < 0.01); reoperation (2.15% vs. 1.36%; RR, 1.57; 95%CI, 1.17 to 2.12, p = <0.01); leak (1.76% vs. 1.02%; RR, 1.57; 95%CI, 1.72 to 2.42, *p* < 0.01); and bleeding (1.66% vs. 1.00%; RR, 1.66; 95%CI, 1.7 to 2.34, p < 0.01).

**Conclusions:**

The study shows that single-stage LRYGB and LSG as revisional surgery after gastric banding, are safe in the 30-day observation with an acceptable complication rate and low mortality. However, conversion to LRYGB increased the risk of perioperative complications.

## Introduction

Laparoscopic adjustable gastric banding (LAGB) was a popular bariatric procedure in the late 1990s and early 2000s [1] However, studies with long-term follow-up revealed poor weight loss outcomes and serious complications, including band erosion, slippage, and gastric pouch enlargement. [2] The majority of patients after LAGB required band removal or conversion to another bariatric procedure due to weight regain and complications. [3–7] Reoperation, after failed gastric banding, is a controversial topic. Band removal with conversion to gastric bypass or sleeve gastrectomy in a single-step procedure is one treatment option. In the analysis of MBSAQIP Participant User File (PUF) from 2015 revealed that conversion to sleeve gastrectomy might be a safer approach than gastric bypass surgery. [8] The observation was surprising because most surgeons advocate conversion to gastric bypass. We decided to replicate the study utilizing MBSAQIP data from 2015 to 2018 and addressing critical methodological flaws.

This study aimed to assess the safety of revisional surgery to laparoscopic sleeve gastrectomy (LSG) compared to laparoscopic Roux-Y gastric bypass (LRYGB) after failed laparoscopic adjustable gastric banding (LAGB) based on MBSAQIP Participant User File from 2015 to 2018.

## Materials and Methods

### Study Design

This study was a retrospective registry-based analysis of patients who underwent a one-stage conversion of LAGB to LSG (Conv-LSG) or LRYGB (Conv-LRYGB). All surgeries were performed between January 1, 2015, and December 31, 2018, at centers participating in the Metabolic and Bariatric Surgery Accreditation and Quality Improvement Program (MBSAQIP). The MBSAQIP prospectively collects data on many variables, including standardized demographics, preoperative comorbidities, laboratory values, and 30-day postoperative mortality and morbidity outcomes for patients undergoing bariatric treatment in participating hospitals in the United States and Canada. [9] The definitions of variables are available in the official MBSAQIP manual. [10]

### Study Population – Strategy Selection

**FIG. 1:**
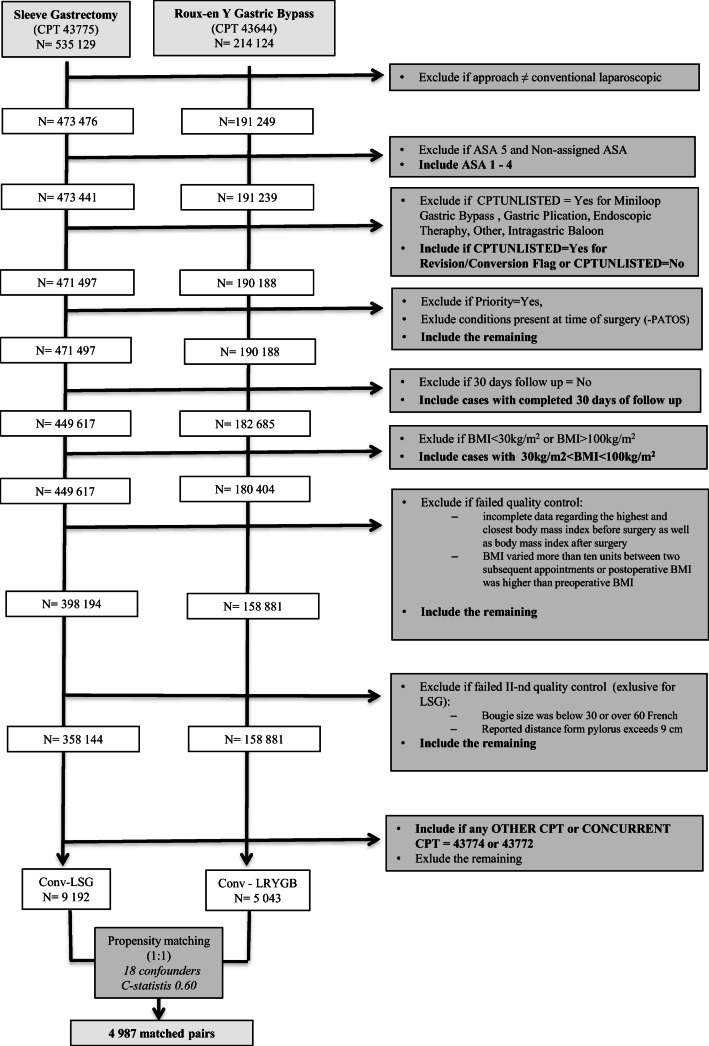
**Flow chart of the study**

Figure 1 shows the Strategy for Case Selection. Data analysis was performed using current recommendations and checklists for large surgical datasets. [11–13] In the first step, we identify all cases of patients who underwent sleeve gastrectomy or Roux-en-Y gastric bypass (as indicated by the use of 43,775 or 43,644 as the principal current procedural terminology [CPT] code for surgery type, according to the MBSAQIP PUF manual). In the next steps, we excluded cases whose initial surgical approach was listed as other than laparoscopic conventional. Other exclusions were: American Society of Anesthesiologists (ASA) class of 5 or missing, cases with additional unlisted CPT codes (for Miniloop Gastric Bypass, Gastric Plication, Endoscopic Therapy, Other, Intragastric Baloon), emergency cases, conditions present at the time of surgery, lack of 30-day follow-up, and BMI below 30 or over 100 kg/m2.

Additional validation & quality tests were performed on two cohorts to remove cases with the following criteria from the PUF 2015, PUF 2016, PUF 2017, and PUF 2018 datasets.

• Records with **incomplete data** regarding the highest and closest BMI before or after surgery.

• **BMI variation of more than ten units** between two subsequent postoperative appointments.

• **Postoperative BMI > preoperative BMI.**

• [only LSG patients] Reported **bougie size below 30 or over 60 French**. Bougie size range described in the literature is between 30 and 60 French. [14]

• [only LSG patients] Reported **distance from pylorus exceeding 9 cm**. Distance >10 cm was not described in the literature. [15]

Observations with missing data were excluded from the analysis.

Finally, all observations with at least one code for AGB removal [43,774, 43,772] listed as “Other CPT” or Concurrent CPT codes” were identified as cases of adjustable gastric band removal (AGB) with one stage conversion to Laparoscopic Sleeve Gastrectomy (Conv-LSG) or Laparoscopic Roux-Y Gastric Bypass (Conv-LRYGB). Conv-LRYGB cases were matched (1:1) with Conv-LSG patients using propensity scoring to control for potential confounding.

### Outcomes

The primary outcome of the study was **all-cause mortality**. Secondary outcomes included:

• **Operative time (OR).**

• **Length of hospital stay (LoS).**

• **Emergency department (ED) visits within 30 days postoperative.**

• **30-day readmission.**

• **30-day intervention.**

• **30-day reoperation.**

• **Leak rate** (defined as drain present >30 days, organ space surgical site infection, leak-related 30-day readmission, or leak-related 30-day reoperation or intervention),

• **Bleeding event** (defined as bleed-related 30-day readmission, bleed-related 30-day reoperation, or transfusion required within 72 h postoperatively),

• **30-day morbidity.**

• Including unplanned admission rate to the Intensive Care Unit within 30 days, pulmonary embolism, space surgical site infections, progressive renal insufficiency, postoperative sepsis, unplanned intubation, postoperative urinary tract infections, vein thrombosis requiring therapy**,** acute renal failure, postoperative cardiac arrest requiring CPR, coma over 24 h, stroke or cerebrovascular accident, postoperative deep incisional surgical site infections, postoperative myocardial infarction, postoperative ventilation, intraoperative nerve injury, pneumonia, postoperative septic shock, unplanned intubation, *Clostridium difficile* infection, and wound disruption.

### Statistical Analysis

Propensity scoring was used to minimize selection bias. The matching procedure was based on the probability of having conversion to LRYGB. Cases were matched with controls by 18 variables. The matching was done using a 1:1 greedy-matching algorithm, with a caliper of 0.05 standard deviation of the logit of the propensity score. [16] We assessed standardized differences for all the baseline covariates after matching to check the postmatch balance. Standardized differences below 0.1 for a given covariate indicate a small imbalance. In the matched cohort, continuous outcomes were analyzed using the paired t-test or Wilcoxon signed ranks test. Dichotomous outcomes analyzed using McNemar’s analysis or Cochran–Mantel–Haenszel test. A description of effect estimates (risk ratio, RR) and 95% confidence interval (95% CI) was reported for category outcomes. The mean difference (or median) with 95% CI was reported for continuous variables. The analysis was performed using SAS® software, University Edition (SAS 9.4 Institute Inc., Cary, NC, USA).

## Results

A total of 14,235 cases were used in propensity score matching (Fig. 1). Table 1 presents the baseline characteristics of analyzed patients. Patients who were converted to LRYGB were more likely to have diabetes, sleep apnea, hypertension, hyperlipidemia, GERD, and had higher BMI. After matching, 4987 pairs were included in the final analysis. The C-statistic for the model was 0.604. The standardized differences were < 0.1 for all variables, indicating a lack of significant differences between the two groups. (Table 2).

**Table 1:** Demographic characteristics

Characteristic	Original cohort			Matched cohort		
	Conv-LRYGB *N* = 5043	Conv-LSG *N* = 9192	*p* value	Conv-LRYGB *N* = 4987	Conv-LSG N = 4987	p value
	Mean (SD) or % / Median Q1 and Q3			Mean (SD) or % / Median Q1 and Q3		
Age (years)	48.65 ± 10.78	48.27 ± 10.84	0.053	48.62 ± 10.77	48.51 ± 10.98	0.671
BMI (kg/m2)	43.35 ± 6.81	42.03 ± 6.85	<0.001	43.27 ± 6.75	43.33 ± 7.32	0.622
Sex (female)	85.52	82.96	<0.001	85.42	85.42	0.977
Race (white)	78.78	76.30	<0.001	78.70	78.93	0.799
GERD	37.99	31.20	<0.001	37.58	36.96	0.502
Pre-Op Obstructive Sleep Apnea*	32.58	27.08	<0.001	32.30	32.30	–
Pre-Op Hypertension	47.89	44.88	<0.001	47.66	47.02	0.525
Pre-Op Hyperlipidemia	24.89	21.78	<0.001	24.42	23.64	0.314
Pre-Op Diabetes Mellitus	21.55	16.22	<0.001	21.13	21.09	0.979
Current Smoker within One Year	5.55	6.85	<0.023	5.59	5.25	0.470
Preoperative Renal Insufficiency	0.50	0.23	0.009	0.40	0.34	0.736
History of Severe Chronic Obstructive Pulmonary Disease (COPD)	1.13	1.06	0.187	1.26	1.14	0.645
Pre-Op Vein Thrombosis Requiring Therapy	2.24	1.43	<0.001	2.15	1.86	0.346
History of Pulmonary Embolism	1.17	1.33	0.436	1.16	1.24	0.779
The medical specialty of the physician performing the principal operative procedure *(bariatric surgeon)*	72.91	73.72	0.302	73.21	73.69	0.604
Steroid/Immunosuppressant Use for a Chronic Condition	1.43	2.06	0.007	1.44	1.26	
ASA Class			<0.001			0.302
I	0.20	0.39		0.20	0.14	
II	22.93	28.02		23.16	23.34	
III	73.75	68.68		73.57	73.57	
IV	3.12	2.91		3.07	2.95	
V	–					

PCI, Percutaneous coronary interventions; PTCA, Percutaneous transluminal coronary angioplasty

*- No discordant pairs, p value was not calculated

**Table 2:** Standardized differences in propensity score-matched sample

Variable	d
Age *(years)*	0.010
Body Mass Index *(kg/m2)*	0.009
Sex (female)	0.001
Race *(white)*	0.005
Pre-Op Hypertension	0.013
Pre-Op Diabetes Mellitus	0.001
Pre-Op Hyperlipidemia	0.018
Pre-Op Obstructive Sleep Apnea	0.011
Gastroesophageal Reflux Disease (GERD) Requiring Medication (within 30 days before surgery)	0.013
Previous Percutaneous coronary interventions (PCI) / Percutaneous transluminal coronary angioplasty (PTCA)	0.013
Pre-Op Vein Thrombosis Requiring Therapy	0.020
History of Pulmonary Embolism	0.007
Preoperative Renal Insufficiency	0.010
History of Severe Chronic Obstructive Pulmonary Disease (COPD)	0.011
ASA Class	0.004
Steroid/Immunosuppressant Use for a Chronic Condition	0.016
Current Smoker within One Year	0.015
The medical specialty of the physician performing the principal operative procedure *(bariatric surgeon)*	0.011

### Outcomes of Interest

The results are presented in Table 3. Mortality was very low and comparable between Conv-LRYGB and Conv-LSG (0.02% vs. 0.06%; relative risk [RR], 0.33 95% confidence interval [CI], 0.03–3.20, *p* = 0.317). The most likely reason for death was listed as “*Other”* in all cases. Conv-LRYGB cases had significantly longer operative time (mean difference, MD 53.78 [±80.18] minutes, *p* < 0.001). Length of hospital stay (MD 0.41 [±2.55] days, p < 0.001) and the conversion rate (0.56% vs. 0.40%; RR, 1.40; 95%CI, 0.79–2.48, *p* = 0.248) were comparable between groups. Conversion to LRYGB increased risk for ED visits (7.74% vs. 4.71%; RR, 1.64; 95%CI, 1.33–2.03, *p* < 0.001), readmission (6.16% vs. 3.77%; RR, 1.63; 95%CI, 01.37–1.94, p < 0.001), reoperations (2.15% vs. 1.36%; RR, 1.57; 95%CI, 01.17–2.12, *p* = 0.003), leak (1.76% vs. 1.02%; RR, 1.72; 95%CI, 1.23–2.42, p < 0.001), bleeding (1.66% vs. 1.00%; RR, 1.66; 95%CI, 1.17–2.34, *p* = 0.004) and any medical complication (4.97% vs. 2.35%; RR, 2.12; 95%CI, 1.70–2.63, p < 0.001). Top ten most likely reasons for readmission and reoperation are presented in Tables 4 and 5.

**Table 3:** Main outcomes of interes

**Outcomes**	**CONV – LRYGB** **N = 4987**		**CONV – LSG** ***N*** **= 4897**		**Mean difference ± SD**	**P -value**
**Continuous**	**Mean ± SD**		**Mean ± SD**			
Length of bariatric procedure (min)	162.28 ± 64.94		108.45 ± 46.78		53.78 ± 80.18	<0.001
Length of hospital stay (days)	2.05 ± 1.53		1.63 ± 2.04		0.41 ± 2.55	1.000
**Categorical**	**%**	***N***	**%**	***N***	**Risk Ratio [95%CI]**	**P -value**
Death within 30 days	0.02%	*1*	0.06%	*3*	RR: 0.33 [0.03–3.20]	0.317
Conversions to open approach	0.56%	*28*	0.40%	*20*	RR: 1.40 [0.79–2.48]	0.248
Emergency department (ED) visits within the 30 days postoperative	7.74%	*217*	4.71%	*132*	RR: 1.64 [1.33–2.03]	<0.001
Inpatient readmission(s) by midnight of POD 30	6.16%	307	3.77%	188	RR: 1.63 [1.37–1.94]	<0.01
Interventions performed within 30 days	3.19%	*159*	1,32%	*66*	RR: 2.41 [1.82–3.19]	<0.001
Reoperations performed within 30 days	2.15%	*107*	1.36%	*68*	RR: 1.57 [1.17–2.12]	0.003
Leak within 30 days	1.76%	*88*	1.02%	*55*	RR: 1.72 [1.23–2.42]	0.001
Bleeding within 30 days	1.66%	*83*	1.00%	*50*	RR: 1.66 [1.17–2.34]	0.004
Any medical complications within 30 days	4.97%	*248*	2.35%	*117*	RR: 2.12 [1.70–2.63]	<0.001

**Table 4:** Top 10 the most likely reasons for **readmission**

**TOP 10 for Readmission**					
**CONV-LRYGB** ***N*** **= 307**			**CONV-LSG** ***N*** **= 188**		
**Reason**	**N**	**%**	**Reason**	**N**	**%**
1. Nausea and Vomiting, Fluid, Electrolyte, or Nutritional Depletion	60	19.54	1. Nausea and Vomiting, Fluid, Electrolyte, or Nutritional Depletion	45	23.94
2. Abdominal Pain, Not Otherwise Specified	46	14.98	2. Other	25	13.30
3. Intestinal Obstruction	33	10.75	3. Anastomotic/Staple Line Leak	24	12.77
4. Other	29	9.45	4.Abdominal Pain, Not Otherwise Specified	21	11.17
5. Anastomotic/Staple Line Leak	16	5.21	5. Other Abdominal Sepsis	12	6.48
6. Bleeding	16	5.21	6. Strictures / Stomal Obstruction	12	6.38
7. Other Abdominal Sepsis	14	4.56	7. Vein Thrombosis Requiring Therapy	11	5.85
8. Wound infection / Evisceration	14	4.56	8. Bleeding	7	3.72
9. Strictures / Stomal Obstruction	12	3.91	9. Pneumonia	5	2.66
10. Infection/Fever	11	3.58	10. Pulmonary Embolism	5	2.66

Top 10 for LRYGB = 81,76% / LSG = 88.83%

**Table 5:** Top 10 the most likely reasons for **reoperation**

**TOP 10 for Reoperation**					
**CONV-LRYGB** ***N*** **= 107**			**CONV-LSG** ***N*** **= 68**		
**Reason**	**N**	**%**	**Reason**	**N**	**%**
1. Intestinal Obstruction	31	28.97	1. Anastomotic/Staple Line Leak	17	25.00
2. Anastomotic/Staple Line Leak	13	12.15	2. Other	14	20.59
3. Bleeding	13	12.15	3. Bleeding	13	19.12
4. Other	13	12.15	4. Strictures / Stomal Obstruction	7	10.29
5. Incisional Hernia	7	6.54	5. Other Abdominal Sepsis	5	7.35
6. Wound Infection / Evisceration	6	5.61	6. Wound Infection / Evisceration	4	5.88
7. Abdominal Pain, Not Otherwise Specified	4	3.74	7. Intestinal Obstruction	2	2.94
8. Internal Hernia	4	3.74	8. Nausea and Vomiting, Fluid, Electrolyte, or Nutritional Depletion	2	2.93
9. Other Abdominal Sepsis	4	3.74	9. Abdominal Pain, Not Otherwise Specified	1	1.47
10. Planned Surgery	3	2.80	10. GI perforation	1	1.47

Top 10 for LRYGB = 91,59% / LSG = 97.06%

## Discussion

After a decade of popularity, adjustable gastric banding almost vanished. The reasons were poor weight loss outcomes and late severe complications, including band erosion, slippage, and gastric pouch enlargement. [2] Revision of failed gastric banding has become a common practice. [3–7] Choosing the next bariatric procedure is a controversial topic. Conversion to Roux-en Y gastric bypass and sleeve gastrectomy is the most common. It can be done concomitantly or as a two-stage procedure. The single-stage approach has a significant advantage - require fewer total surgeries. Thus, it is recognized as less demanding for patients. However, it is demanding for a surgeon, who needs to take down the band and fibrosis capsule before proceeding with the next step. Moreover, firing staples through the band related gastric inflammation does not seem to be the best idea. The meta-analysis by Zadeh at el. revealed that a one-stage approach was favored in the Roux-en-Y subgroup, but two-stage conversions appeared to have a lower risk of a leak in the sleeve gastrectomy subgroup. [17]

The difference was explained by the involvement of different anatomy regions in each surgery. In conversion to Roux-Y gastric bypass, a pouch creation and gastro-jejunal anastomosis can be done above the band’s place. In sleeve gastrectomy, the portion of the stomach compressed by the gastric band is usually transected by the stapler. The two-stage approach gives the time needed to resolve the inflammation and thus reduces the staple line leak risk. For this reason, surgeons are afraid of doing a conversion to LSG as a single-stage procedure.

The analysis of MBSAQIP data from 2015 was the first study showing the opposite. Surprisingly, the single-stage conversion of failed gastric banding was associated with more significant morbidity and higher complication rates when converted to LRYGB versus LSG. These differences were notable regarding bleeding events, 30-day reoperation, 30-day readmission, operative time, and hospital stay. [8]

The conclusion was far from popular belief. Despite the utilization of a sizeable medical registry, the analysis did not achieve sufficient power to detect a significant leak rate.

We decided to replicate the study utilizing MBSAQIP data from 2015 to 2018 and addressing critical methodological flaws. Several issues make the present analysis unique. First, using datasets from 2015 to 2018, we obtained a large sample size. This sample size improved the power of our analysis significantly and was sufficient to detect meaningful differences. Second, Noyes et al. and Papasavas et al. stated various data quality issues in MBSAQIP datasets. [18, 19]

For this reason, we improved the strategy for the study population selection and performed additional quality checks. Third, we applied propensity score matching using the greedy algorithm. The algorithm has an important feature – it does not assign the same control subjects for different cases. At present, this method guarantees the best possible matched set. We also decided to investigate the magnitude of effect size than interpret null-hypothesis significance testing. [20] Lastly, we analyzed the most likely reasons for readmission and reoperation, which shed more light on this challenging topic. The presented analysis revealed that conversion to LRYGB did not increase the risk of 30-day death. The tendency was opposite but not statistically significant. The most likely reason for death was stated as *“Other”* for all cases. Overall, the conversion of failed gastric banding to either LRYGB or LSG was safe with acceptable low mortality.

Our study supported previous observations. First, the single-stage conversion is safe, and the overall mortality is very low. Second, the one-stage revision sleeve gastrectomy compared to a one-stage Roux-Y gastric bypass after failed gastric banding had a better safety profile in the short term. Our study is the first showing a higher risk for an anastomotic leak, 30-day intervention, and emergency department visits in the group converted to LRYGB. The need for conversion to open in our study was low. The analysis of most likely reasons for reoperation showed that each procedure has its profile of complication. In the group of patients after conversion to LRYGB, most reoperations were due to intestinal obstruction, whereas in Conv-LSG, the most common reason was a staple line leak.

It is essential to highlight that although conversion LAGB to LRYGB increased from 57% to 141% reoperation rate, it does not increase the overall mortality. A possible reason is a fact that complications after LRYGB are easier manageable. Small bowel obstruction (SBO) after LRYGB is commonly reported in the literature. Internal hernia and adhesions are responsible for most cases for SBO. Operative treatment remains the definitive standard and should not be delayed. [21] On the other hand, staple line after sleeve gastrectomy is challenging to manage. The occurrence of this complication increases the mortality significantly. [22] Treatment requires time. According to Sakran, the median time to staple line leak closure is 40 days. [23] It is much more than the median time reported for leaks after gastric bypass surgery, 13 days. [24]

Likely, the 30-day observation period stated in the MBSAQIP can not capture the outcome in the cases where the leak after conversion to LSG was present regarding reoperation, intervention, and death. Even though the leak was the most likely reason for reoperation in the Conv-LSG group, the incidence was lower than in Conv-LRYGB patients. In our analysis, laparoscopic sleeve gastrectomy after gastric banding showed a leakage rate of 1.02%, which was higher than reported from primary LSG in the MBASQIP database – ranging from 0.6 to 0.9%. [15, 25]

In our study, patients converted to LRYGB had an anastomotic leakage percentage of 1.76%. It corresponded to the analysis from 2015 and was higher than the reported rate for primary LRYGB in MBSAQIP analysis (0.6%). [26]

Our study showed that conv-LRYGB increased the risk of short term complications in comparison to conv-LSG significantly. However, the effect size was mainly moderate, and it is difficult to determine if both procedures are comparable in terms of long-term complications because there is not enough data.

What is more, reports are indicating the significant benefit of conversion to LRYGB. The recent meta-analysis showed that conversion to LRYGB was associated with better weight loss at 12 and 24 months compared to conversion to LSG. [27]

Our study’s essential message is the following: both conversions of failed gastric banding have an excellent safety profile with acceptable complications rate. Although conversion to Laparoscopic Roux-Y gastric bypass increased the risk for short-term complications, the effect size is moderate. In this situation, the risk profile should not outweigh the weight loss benefits.

Our study has a few significant limitations. First, the MBSAQIP database is observational. The association between adverse events should be tested prospectively in a controlled environment to evaluate a potential causal relationship. However, considering the low rate of adverse events, it would be challenging to conduct a randomized control study with enough power to show a difference. Second, we could not assess the efficacy of conversions because of the lack of data on weight loss outcomes and comorbidities improvement. MBSAQIP database does not provide sufficient data on significant intraoperative variations in techniques. For this reason, we were not able to assess some essential technical details. Similarly, significant factors that could affect risk profile, such as operative findings and indications, were also not included.

## Conclusion

Our study shows that single-stage laparoscopic Roux-Y gastric bypass and laparoscopic sleeve gastrectomy as revisional surgery after gastric banding, are safe in the 30-day observation with an acceptable complication rate and low mortality. The conversion to LRYGB is more demanding and increases the risk of leak rate, bleeding events, reoperation, intervention, readmission, longer operative time and more extended hospital stay, emergency department visits, and morbidity. However, the effect size for the increase in risk was moderate. The essential message is that increase in complications rate did not result in higher mortality. The decision about the type of procedure should be based on individual patient’s risks-benefits assessment.
